# Scientists from small countries on the hunt for scientometric figures – global indicators and local finances

**DOI:** 10.3325/cmj.2024.65.73

**Published:** 2024-04

**Authors:** Damir Stanzer

**Affiliations:** Department of Food Engineering, Faculty of Food Technology and Biotechnology, University of Zagreb, Zagreb, Croatia

**Figure 1:**
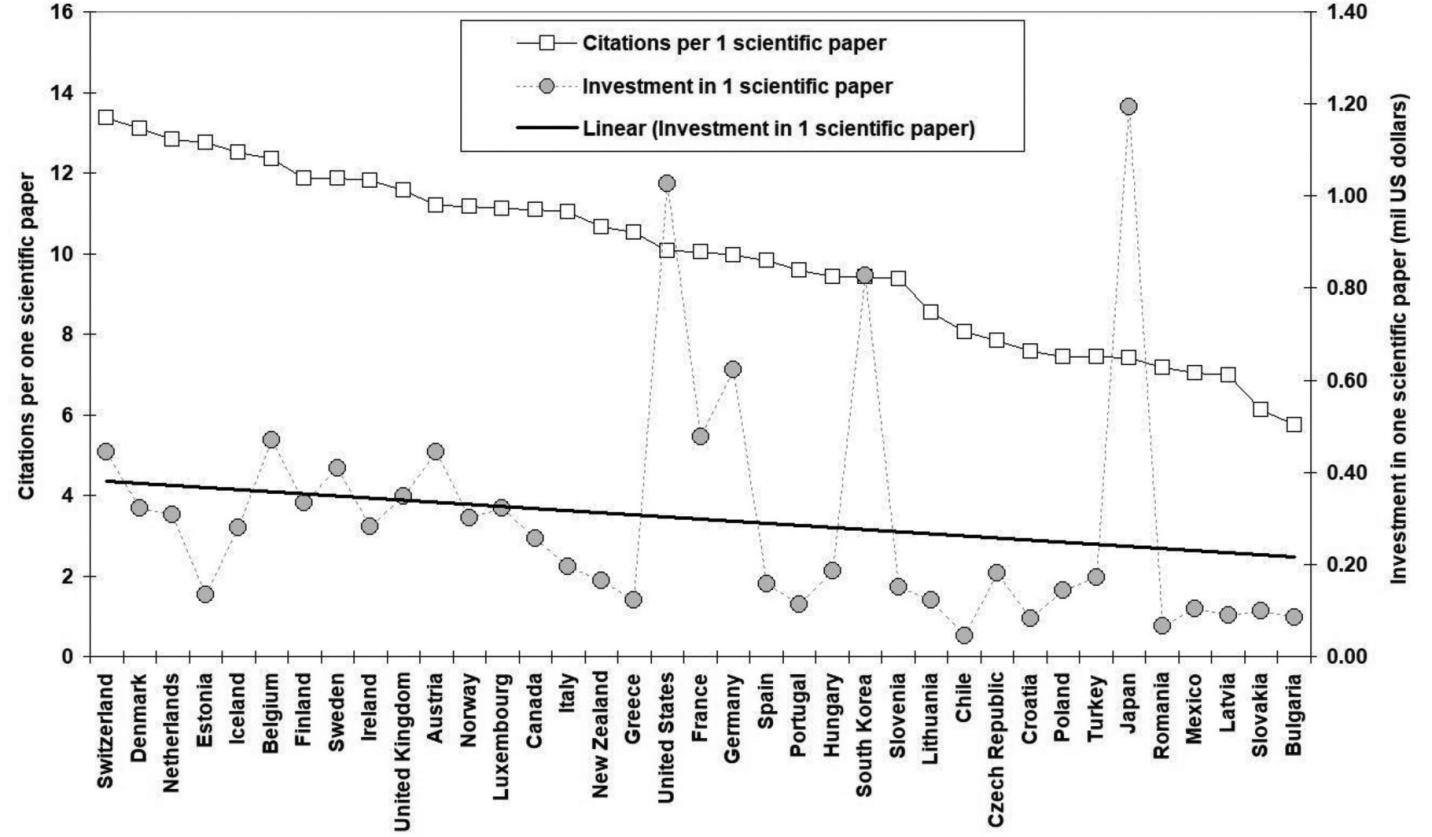
The number of scientific articles published in 2020 that are indexed in the Scopus database (review: Scimago, April 2024) in relation to the number of researchers in the respective country in that year (data: World Bank). The countries included are those of the Organisation for Economic Co-operation and Development and the European Union. The countries clearly differ in the productivity of researchers. For a more serious analysis, however, the proportion of researchers in the articles published in international collaborations should be calculated. Nevertheless, the figure indicates different pressures that the scientific systems in various countries exert on local scientists to publish as many scientific articles as possible.

The shortcomings of scientometrics have been known for a long time. Dissatisfaction with its use and misuse in evaluating scientists and scientific institutions is as old as the discipline itself. And yet, scientometrics has persisted to this day. All this counting of publications, citations, journal impact factors, and H-indexes is obviously detrimental to real science. It risks turning an indicator into a target (Figure 1) and replacing the quality of scientific discoveries by the ability of a scientist or institution to achieve certain numerical targets.

We scientists, with some valuable exceptions, have mostly tacitly resigned ourselves to this chase. This is hardly surprising as the alternative is purely subjective evaluation, ie, having our quality assessed by a committee of colleagues with their various biases. But numerical measurement of quality in science was not invented by scientists. It was invented for the interests of private journal publishers, and later embraced by financiers in the systems of science (politicians or bureaucrats).

**Figure 2:**
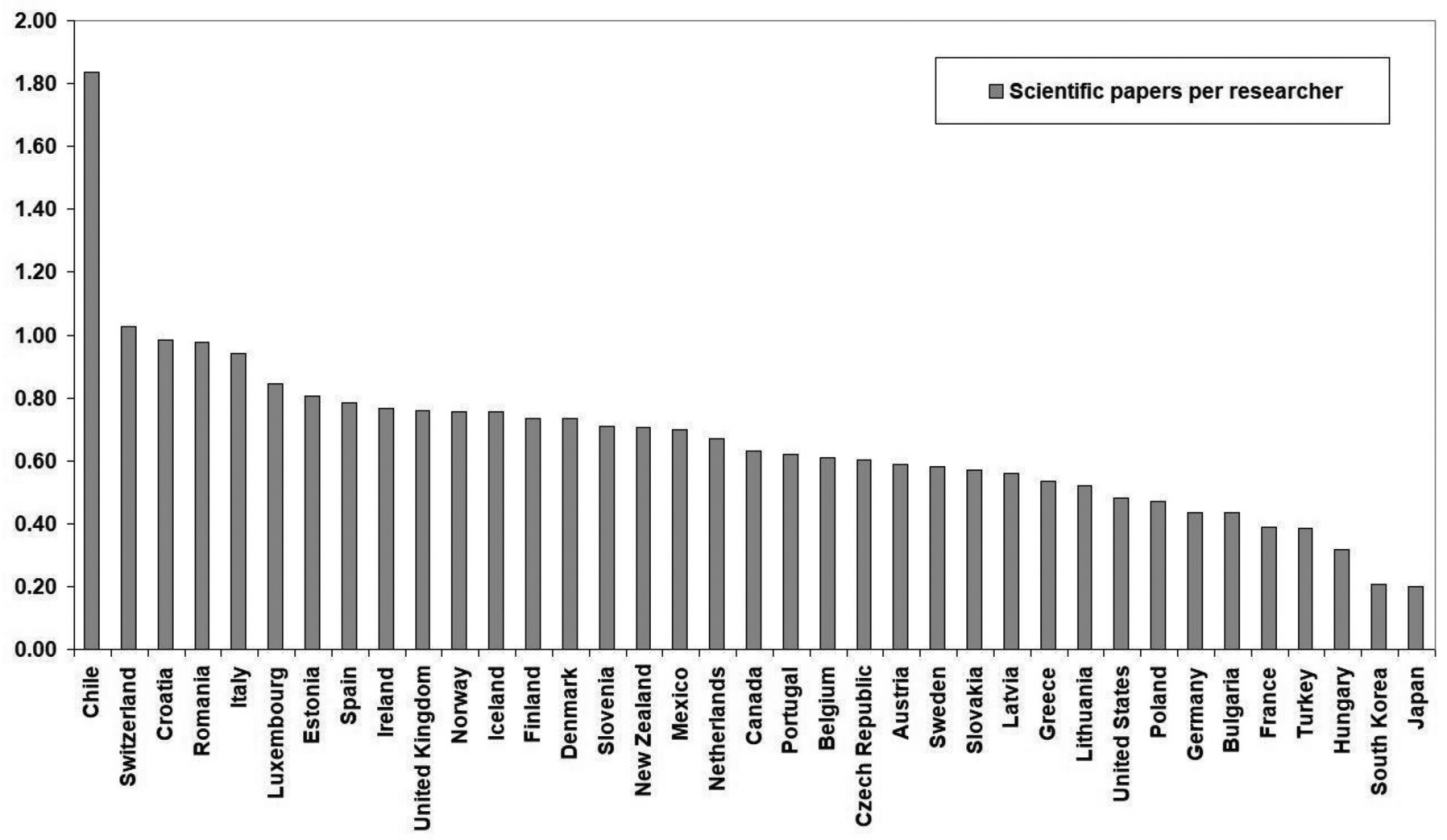
The average number of citations of each scientific article from 2020 indexed in the Scopus database (review: Scimago, April 2024) in relation to the “average price of an article” obtained by dividing the total domestic investment in research and development in 2020 (data: World Bank) by the number of articles. The countries included are those of the Organisation for Economic Co-operation and Development (OECD) and the European Union. If one naively equates the average number of citations per article with “quality” (which is certainly not correct in this case due to the different shares of various scientific fields in the individual countries, which are differently citable), one can conclude from the trend line of the article price that the number of average citations per article decreases almost proportionally to the decrease in the amount of money invested in the article. Logical – more money leads to more citations. However, the matter is much more complex. The article prices in individual countries considerably deviate from the trend line, and, obviously, richer systems (eg, USA, South Korea, Japan) invest significantly more and weaker systems (eg, Chile, Croatia, or Romania) significantly less money in the publication of one article than would be expected given the average citation of the article. The reasons for these deviations are manifold and certainly include the non-scientific part of research and development in richer countries, the influence of the article topic on citations and self-citations caused by the number of researchers in larger scientific communities, as well as international collaborations - “import” of citations by smaller countries (visible in the example of Estonia).

Rather than achieving the much-desired scientific cooperation between scientists, this hunt for numbers has managed to stir competition. James Carville's classic quote, “It's economy stupid!” does not apply only to politics, but also to scientific systems. Indeed, there is money at the beginning and end of every scientific undertaking. The input or output of science is a number. The easiest way to attribute this numerical input (funding) to a scientist is based on another number – the number of articles, citations, etc. In this respect, the scientific community behaves quite strangely. Not only do these supposedly brightest minds in the world tacitly agree to use such an imperfect and unscientific method to measure their own quality, they even agree to use it incorrectly. It is strange, for example, that the world's universities agree to participate in failed “university rankings,” with numerical measures of “survey reputation” or the “number of Nobel laureates as an indicator of the quality of the institution.” But no less strange is the use of the number of articles published and the number of citations, as well as various derivations of these parameters, as ways to measure scientific quality. These seemingly solid indicators, which are real numbers, are taken out of context and often misinterpreted. They are regularly used outside of a much more complex picture, while other numbers that really need to be included in the calculation are forgotten. Some of these numbers are the number of researchers and the absolute amounts of funding, which are just as hard numbers as the number of articles and citations. Does it make sense to quantify the output without quantifying the input (Figure 2)? The number of people producing scientific output is often overlooked. For example, what does it mean for the scientific productivity of that particular country if there are 358 researchers per million inhabitants in Mexico, 941 in Romania, around 2002 in Croatia, and around 7700 in Sweden and Denmark? Or how can we compare the quality of a researcher who is the sole author of a scientific article with that of a researcher who is one of 5000 co-authors? Is it possible to compare institutions with 20 historians with those with 350 physicists or physicians by using the number of publications and citations? Do Scopus or Web of Science, which divide science into subject areas, solve this problem completely, or have we in this way only assuaged consciences and somewhat reduced the problem that still exists? How about a competitive comparison between physicians, physicists, or sociologists who publish articles in small teams and those who work on large collaborative projects? What does the H-index as a hard number tell us about this, and what does the institution's total number of articles and citations?

Even in the strongest, well-funded scientific communities in the world, scientists are constantly living in existential angst and wage battles for funding against their colleagues. Scientists in small and poorly funded systems are in an even graver financial situation. But often, the managers of these systems supposedly do not understand the scientometric numbers they like to use. They demand a global output from scientists and provide them with local funding. The share of the gross domestic product (GDP) invested in research and development, an indicator sometimes considered in scientometric theory (but never in practice), is not actually an input indicator. It roughly indicates the state of the country, the political will, and the strength of the private sector to invest in research. The real input is real money – the amount of euros or US dollars. If a scientist from Croatia buys a high-performance liquid chromatography device or subscribes to a scientific journal, the price for her is not expressed in terms of “percentage of Croatian GDP,” but is the same as that paid by a scientist from the US or Australia. The actual financial outlay is therefore the absolute amount of euros or US dollars available to the scientist to produce a certain output – in an ideal world, a scientific discovery. In the real world, this money is used to produce a scientific article in the best-ranked journal with as many citations as possible.

If we divide the total investment in research and development by the number of researchers in the country, we see that a researcher in the United States has on average 495 thousand US dollars per year (2020) as input for the production of scientific numbers, in Switzerland 455, in Luxembourg 273, in Ireland 207, and in Iceland 210 thousand, while in the Czech Republic this amount is 109, in Estonia 108, in Slovenia 107, in Croatia 80, and in Bulgaria only 36 thousand US dollars! National science funding agencies provide different amounts of money to teams of researchers: 500 thousand euros for a three-year scientific project by national public funding agencies in Germany, Slovenia, Czech Republic, and Poland, but only 200 thousand euros per project in Croatia or 100 thousand in Bulgaria! Where can politicians expect more scientific papers in better rated journals (Figure 1)? Moreover, the number of citations is directly related to the open access or closed access publication of the article – publication of an article in open access costs money. For financially weak systems, the money they have to transfer to strong private publishers makes up a fairly large proportion of the overall funding of science. In this case, less money means fewer citations, and this is not necessarily related to the quality of the science. Moreover, the alleged quality assessment based on the number of articles and citations is complicated by international cooperation. Scientists from small countries achieve the number of articles and citations by collaborating with scientific teams from much stronger countries, which is a direct “import” of input and output. The smaller the country, the greater the proportion of foreign quality that is considered domestic. Managers in small communities almost cynically instruct their scientists to collaborate with scientists from stronger countries to achieve this import and transfer of funds, and then count and evaluate the scientometric figures thus obtained as domestic results when assessing domestic quality. The ability to join strong international teams becomes the most valued quality of a scientist in this case.

Is this another of the many appeals to abandon scientometrics in line with the Dora Declaration, the Leiden Manifesto, Open Science, and other initiatives against classic scientometrics? Well, we should be realistic. Given the circumstances, a wider range of indicators should be included in the use of scientometrics. Perhaps by putting pressure on the managers of the science system, we can reduce the importance of scientometrics or at least improve it. It is vital to understand the bigger picture, to consider all the numbers (and not just some), to put the numbers into more correct relationships, and then to draw the right conclusions, or at least assumptions about quality, from the right premises. We need to add up the numbers correctly so that 2 and 2 make 4, not 10.

